# Immunologic response and memory T cells in subjects cured of tegumentary leishmaniasis

**DOI:** 10.1186/1471-2334-13-529

**Published:** 2013-11-09

**Authors:** Augusto M Carvalho, Andréa Magalhães, Lucas P Carvalho, Olívia Bacellar, Phillip Scott, Edgar M Carvalho

**Affiliations:** 1 Serviço de Imunologia, Hospital Universitário Prof. Edgard Santos, Universidade Federal da Bahia, Salvador, Rua João das Botas s/n, Canela 40110-160, BA, Brazil; 2 Instituto Nacional de Ciência e Tecnologia em Doenças Tropicais, INCT-DT (CNPq/MCT), Salvador, Bahia, Brazil; 3 Department of Pathobiology, School of Veterinary Medicine, University of Pennsylvania, Philadelphia, PA, USA

**Keywords:** Cured leishmaniasis, IFN-gamma, Effector memory CD4+ T cells

## Abstract

**Background:**

The main clinical forms of tegumentary leishmaniasis are cutaneous leishmaniasis (CL) and mucosal leishmaniasis (ML). *L.braziliensis* infection is characterized by an exaggerated production of IFN-gamma and TNF-alpha, cytokines involved in parasite destruction, but also in the pathology. Maintenance of an antigen-specific immune response may be important for resistance to re-infection and will contribute for vaccine development. In the present work we investigated the immune response in CL and ML cured individuals.

**Methods:**

Participants in the present study included 20 CL and 20 ML patients, who were evaluated prior to, as well as 2 to 15 years after therapy. IFN-gamma, IL-2 and TNF-alpha production were determined by ELISA in supernatants of mononuclear cells stimulated with soluble *L.braziliensis* antigen (SLA). The frequency of memory CD4+ T cell populations was determined by FACS.

**Results:**

Here we show that the majority of CL and ML patients did not produce *in vitro* IFN-gamma in response to SLA after cure. In the cured individuals who responded to SLA, effector memory (CD45RA^-^CCR7^-^) CD4+ T cells were the ones producing IFN-gamma. Because a large percent of CL and ML cured patients lost SLA-induced IFN-gamma production in peripheral blood, we performed *Leishmania* skin test (LST). A positive LST was found in 87.5% and 100% of CL and ML cured individuals, respectively, who did not produce IFN-gamma or IL-2 *in vitro*.

**Conclusion:**

This study shows that in spite of losing *in vitro* antigen-specific response to *Leishmania*, cured CL and ML subjects retain the ability to respond to SLA *in vivo*. These findings indicate that LST, rather than IFN-gamma production, may be a better assessment of lasting immunity to leishmaniasis in human studies, and thus a better tool for assessing immunization after vaccine. Furthermore, in cured individuals which maintains *Leishmania*-specific IFN-gamma production, effector memory CD4+ T cells were the main source of this cytokine.

## Background

Control of *Leishmania* infection relies on cell-mediated immune response, as IFN-gamma induced activation of macrophages is known to be the main mechanism of parasite destruction within these cells. Peripheral blood mononuclear cells (PBMC) from patients with cutaneous leishmaniasis (CL) and mucosal leishmaniasis (ML), due to *L. braziliensis* infection, secrete high levels of TNF-alpha and IFN-gamma that contributes for the control of parasite multiplication and dissemination [1]. However, the inflammatory response observed in these patients is not well regulated and leads to tissue damage and ulcer formation [1,2]. In *L. braziliensis*-infected subjects CD4+ T cells are the main source of IFN-gamma, which is why these cells are the key players in host defense against *Leishmania* infection [3,4]. Moreover, the frequency of memory CD4+ T cells in CL patients is higher than in uninfected controls [3].

Immunological memory is the ability of the immune system to respond fast and better upon antigen exposure than the first exposure to the same antigen. Memory T cells can be maintained long-term, exhibit increased resistant to apoptosis and play a key role in resistance to reinfection and vaccine [5]. The phenotypical and functional characterization of memory T cells, revealed that memory CD4+ T cells constitute a heterogeneous population: CD45RA^-^CCR7^+^ (central memory) and CD45RA^-^CCR7^-^ (effector memory). While effector memory CD4+ T cells can be found in blood and rapidly respond with IFN-gamma production upon re-infection, the central memory ones circulates among lymphoid organs [6], although retains the ability of down-regulate lymph node migration markers expression and become effector cells upon IL-12 production [6,7]. The few studies aimed to understand the dynamics of memory CD4+ T cell populations in mouse model of leishmaniasis showed that early following infection of C57BL/6 mouse, both central and effector memory CD4+ T cells are generated [8,9]. In these mice, *Leishmania* parasites persists for long term and both effector and central memory population long live and mediate protection during re-infection. Differently, a transgenic *L. major* strain, which infected but not survived for a long term in mice, resulted in sterile cure and loss of effector memory CD4+ T cell population, but retained central memory CD4+ T cells. In such case, even in the absence of parasites central memory T cells were able to mediate protection [8,9]. These data suggest that persistence of *Leishmania* parasites is not necessary for the survival of central memory CD4+ T cells and consequently protection to re-infection.

The natural history of human tegumentary leishmaniasis indicates that after lesion resolution individuals acquire long lasting immunity and re-infection with *L. braziliensis* only occurs in the minority of the subjects, as only 5.2% of cured leishmaniasis patients will develop new CL lesions [10]. Indeed, in the Middle East, the practice of immunizing people with a low inoculum of *L. major,* known as “leishmanization”, results in up to 80% protection [11,12].

In the present work we investigated the immune response in CL and ML cured individuals. We found that a sub-group of cured CL and ML individuals have lost circulating *Leishmania* specific Th1 cells, here determined by the absence of SLA-induced IL-2 and IFN-gamma production. Interestingly, despite losing circulating *Leishmani*a reactive Th1 cells, a majority of these individuals retained the ability to respond to *Leishmania* antigens *in vivo* as they remain delayed type hypersensitivity (DTH) positive. Moreover, in these cured subjects who retained the ability to produce IFN-gamma, the source of this cytokine was effector memory CD4+ T cells.

## Methods

### Patients

Twenty CL and twenty ML patients were studied during active disease and 2 to 15 years post therapy. All individuals were recruited at the health post of Corte de Pedra, a region of *L. braziliensis* transmission in southeast of Bahia state, Brazil. All patients were volunteers, informed consent was obtained from all individuals prior to participation in the study, and the research project was approved by the ethical committee of the Federal University of Bahia. The criteria used for the diagnosis of CL and ML were the presence of a typical ulcerated cutaneous or mucosal lesions, associated with a positive DTH test and a positive PCR for *L. braziliensis.* All individuals were treated with pentavalent antimony (20 mg/kg/day) for 20 days for CL and 30 days for ML. The blood for immunological evaluation was drawn before and up to 15 years after therapy, and in both cases it preceded the *Leishmania* skin test (LST).

### Antigen and intradermal skin test

Soluble *Leishmania* antigen (SLA) was prepared as previously described [13], tested for endotoxin using the limulus amebocyte lysate test and used at a concentration of 5 μg/ml. For intradermal skin test, 0,1 ml of *L. braziliensis* antigen was inoculated in the forearm of individuals and induration was determined 48 hours post inoculation. A positive LST was considered when the induration was grater than 5 mm. The antigen used for LST was previously tested in healthy subjects and no induration was observed after 48 hours.

### PBMC culture and ELISA for cytokines

PBMC were isolated from heparin-treated venous blood by ficoll-hypaque gradient centrifugation. After washing three times in 0.9% NaCl, cells were re-suspended in RPMI 1640 culture medium (GIBCO BRL, Grand Island, NY) supplemented with 10% human AB serum, 100 IU/ml of penicillin and 100 μg/ml of streptomycin. Cells were adjusted to 3×10^6^ cells/ml, put in 24-well plates and stimulated with SLA (5 μg/ml). After incubation for 72 hours at 37°C and 5% CO_2_, supernatants were collected and stored at –20°C. The levels of IFN-gamma, TNF-alpha, IL-2 and IL-10 were measured by ELISA (R&D Systems, Minneapolis, MN) sandwich method and the results expressed as pg/ml.

### Flow cytometry

Flow cytometry was performed as previously described [14], Briefly, PBMC (5×10^5^) were stained with fluorochrome-conjugated antibodies for surface markers CD4, CD45RA, CCR7 (e-Bioscience) and fixed by using 2% formaldehyde. For intracellular staining, cells were stimulated with SLA (5 μg/ml) and cultured for 12 hours, plus 8 hours in presence 10 μg/ml of Brefeldin A (Sigma). Cells were than fixed as above, permeabilized with a solution of 0.5% of saponin, and stained for 30 min at 4°C using fluorochrome-conjugated antibodies against IFN-gamma or isotype control antibodies.

Samples were processed on a FACSCantoII flow cytometer (BD Pharmingen), and analysis was performed using FlowJo software (Tree Star). Analysis gates were based live cells, CD4+ T cells and CD45RA negative cells, as indicated in the figure legends.

### Statistical analysis

The Wilcoxon non-parametric paired test was used to assess differences between cytokines levels in the same subjects. Analyses were conducted using Prism (GraphPad Software Inc., San Diego, CA, USA); a P value of <0.05 was considered significant.

## Results

### Demographic and clinical data in CL and CM cured individuals

The age, gender, skin test area (mm^2^), number of series of pentavalent antimony, stage of mucosal disease and time between evaluations are shown in Table 1. The mean age was 23 ± 10 years for cutaneous group and 37 ± 12 years for mucosal patients. Overall, there was a predominance of male individuals (65% and 75%) in CL and ML respectively groups. The median of skin test area was 187 mm^2^ (80 – 625) for CL patients and 300 mm^2^ (56 – 1722) for ML patients. While most individuals with CL (85%) were treated with one serie of pentavalent antimony, 50% of individuals with ML required more than one series. The majority of ML patients were in stage II (superficial ulceration) or stage III (deep ulceration) of mucosal disease [15]. The interval between the evaluations in months was 94 (38 – 161) and 106 (28 – 184) for CL and ML respectively groups.

**Table 1:** Demographic and clinical features of CL and ML cured individuals

	**Cutaneous leishmaniasis (n = 20)**	**Mucosal leishmaniasis (n = 20)**
**Age**	23 (13–48)	37 (12–58)
**Gender (male)**	13 (65%)	15 (75%)
**Skin test area (mm**^**2**^**)**	187 (80–625)	300 (56–1722)
**Series of pentavalent antimony**		
1	17 (85%)	10 (50%)
2	3 (15%)	9 (45%)
3	-	1 (5%)
**Stage of mucosal disease**		
II	-	8 (40%)
III	-	8 (40%)
≥ IV	-	4 (20%)
**Months between evaluations**	94 (38–161)	106 (28–184)

### Loss of SLA-induced IFN-gamma and TNF-alpha production in CL and ML cured individuals

**Figure 1:**
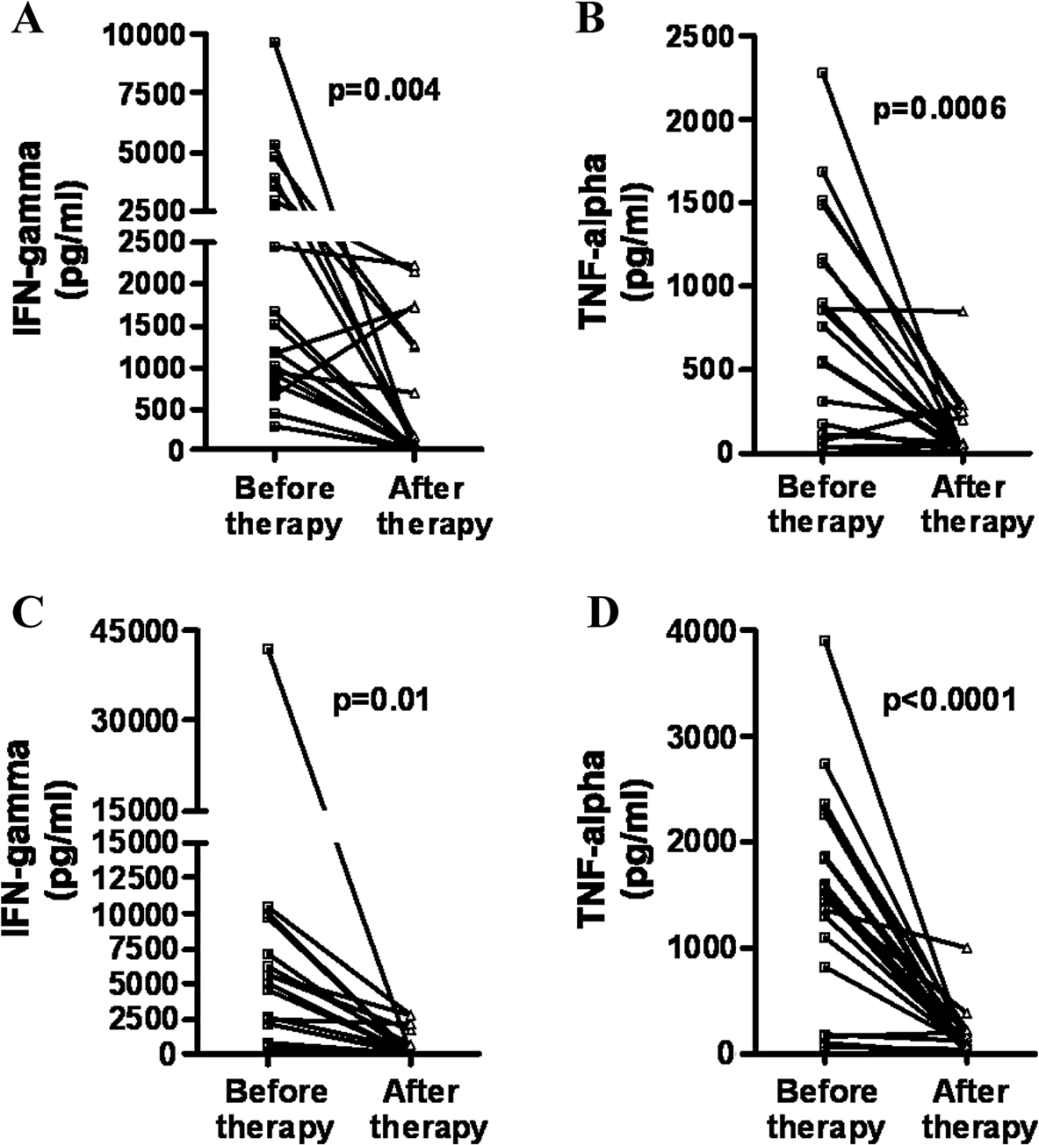
**IFN-gamma and TNF-alpha production in CL and ML patients before and after treatment.** PBMC from patients with CL **(A and B)** and ML **(C and D)** were obtained and stimulated with SLA (5 μg/ml) for 72 hours. IFN-gamma and TNF-alpha levels were determined on culture supernatants by ELISA.

CL and ML patients due to *L. braziliensis* infection mount a strong inflammatory response with high levels of IFN-gamma and TNF-alpha. The levels of *Leishmania*-specific IFN-gamma produced by PBMCs from CL and ML patients significantly decreased after therapy from 1355 pg/ml (282 – 9700) to 78 pg/ml (0 – 2228) and 4759 pg/ml (247 – 41860) to 28 pg/ml (0 – 2769), respectively. Moreover, in 10 (50%) and 11 (55%) of CL and ML cured individuals, respectively, IFN-gamma production was not detected. TNF-alpha levels in CL and ML dropped from 656 pg/ml (0 – 2280) to 24 pg/ml (0 – 847) and 1533 pg/ml (54 – 3895) to 57 pg/ml (0 – 993) respectively (Figure 1). Although in the majority of patients a marked decrease in both IFN-gamma and TNF-alpha was observed, in two patients with CL there was an increase in IFN-gamma after therapy and in one of them TNF-alpha level did not change after therapy. The therapeutic response to antimony in these two cases was similar to the others CL patients. Both of them received only one course of antimony, were cured with less than 90 days after initiation of therapy and the evaluation after therapy was performed 12 years after cure. One of these patients presented the largest cutaneous ulcer. Both patients remained free of disease after 12 years of follow up.

As the time between the evaluations ranged from 28 to 184 months, we determined if the decrease or absence of IFN-gamma production was related to the duration of time between the evaluations. We compared the immune response in CL and ML patients that were evaluated after therapy with 5 years or less, with the group that was evaluated after more than 6 years (Table 2). A significant decrease in IFN-gamma production was observed after therapy in CL subjects that were evaluated with 5 or less years after cure (P = 0.03) or more than 6 years (P = 0.01). A significant reduction in IFN-gamma production in ML patients was also detected in those who were evaluated with or less than 5 years after cure (p = 0.003) or and in ML patients evaluated 6 years after cure (p = 0.001) in comparison with IFN-gamma levels before therapy. In addition to the decreasing or absence of IFN-gamma production, IL-10 levels were undetectable in supernatants of PBMC culture stimulated with *Leishmania* antigen (data not shown). IL-2 production was observed 13 (32.5%) of 40 cured tegumentary leishmaniasis patients (data not shown).There was an association between IL-2 and IFN-gamma production as of the 13 cases who had IL-2 detectable 11 (84.6%) also had IFN-gamma detected. Moreover IL-2 was not produced subjects who did not produce IFN-γ.

**Table 2:** IFN-γ production pre and post therapy in patients with cutaneous and mucosal leishmaniasis

**Disease**	**Time of evaluation after therapy**	**IFN-gamma**		**p**
		**(Median and range)**		**value**
		**Pre therapy**	**Post therapy**	
**Cutaneous**				
**Leishmaniasis**	≤ 5 years	3608 pg/ml	34.5 pg/ml	0.0313
**(N = 6)**		(288 – 9700 pg/ml)	(0 - 2228 pg/ml)	
**Cutaneous**				
**Leishmaniasis**	6 to 13 years	1086 pg/ml	89.5 pg/ml	0.0107
**(N = 14)**		(433 – 3873 pg/ml)	(0 – 2152 pg/ml)	
**Mucosal**				
**Leishmaniasis**	≤ 5 years	4998 pg/ml	12 pg/ml	0.0039
**(N = 9)**		(529 – 41860 pg/ml)	(0 – 2769 pg/ml)	
**Mucosal**				
**Leishmaniasis**	6 to 15 years	4520 pg/ml	64 pg/ml	0.0010
**(N = 11)**		(247 – 9660 pg/ml)	(0 – 2712 pg/ml)	

NOTE: Data are median. (range) of subjects. Statistics analysis was determined by Wilcoxon matched pairs test.

### Evidence of delayed type hypersensitivity (DTH) response in cured CL and ML subjects

Although there is usually a good correlation between DTH and T cells responses *in vitro*, there are evidences of discordant results between *in vivo* and *in vitro* tests of cell-mediated immune response [16,17]. To investigate if individuals cured of CL and ML who did not produce IFN-gamma and IL-2 had evidence of DTH to *Leishmania* antigen, the LST was performed in cured CL and ML subjects without evidence of *in vitro* IFN-gamma production after stimulation with SLA. A positive DTH was found in7/8 (87.5%) and 7/7 (100%) of CL and ML cured individuals, respectively.

### Effector memory CD4+ T cells are the main IFN-gamma secreting cells in CL and ML cured individuals

**Figure 2:**
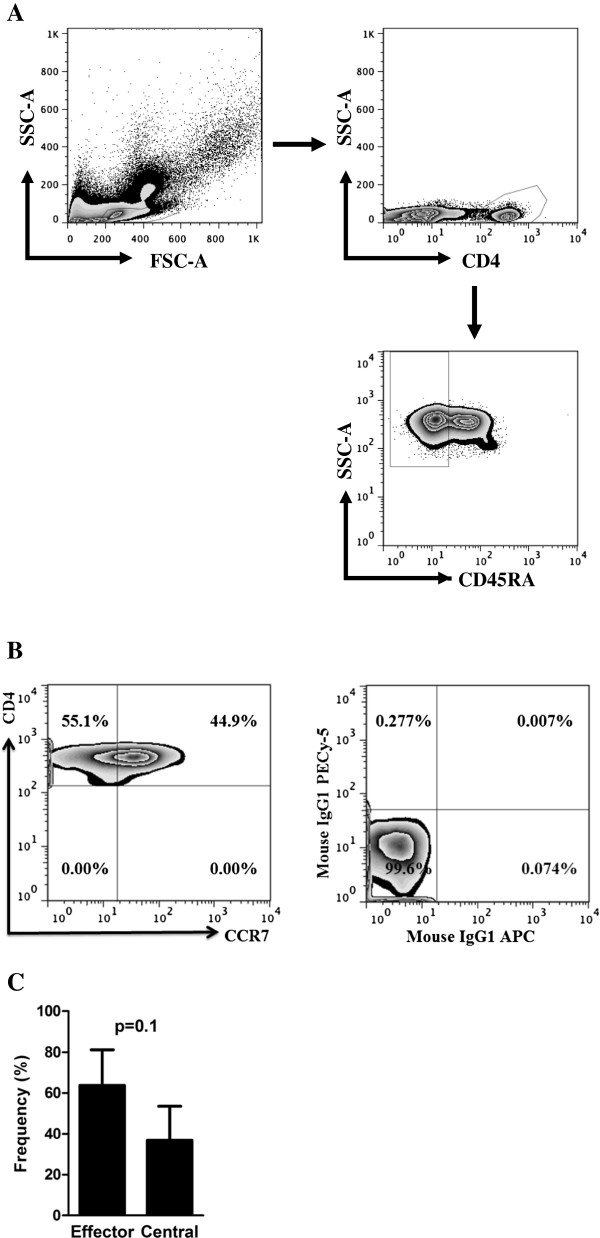
**Percentage of effector and central memory CD4+ T cell populations. A**, gate strategy. **B**, representative plot showing CCR7^-^ (effector memory) or CCR7^+^ (central memory), in a ML cured individual and the isotype control antibodies. **C**, frequency of effector memory and central memory in 7 cured subjects. PBMC were analyzed *ex vivo* gated on CD4^+^ CD45RA^-^.

**Figure 3:**
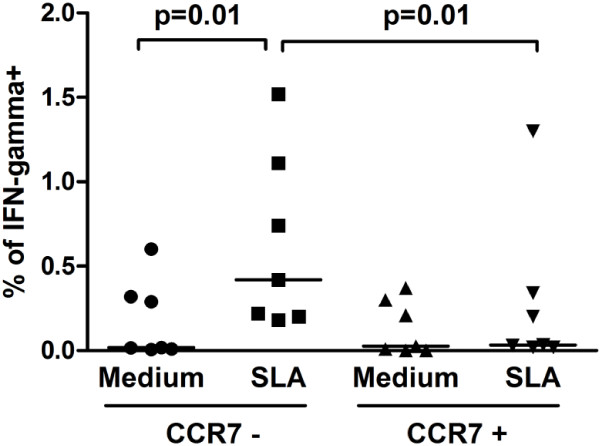
**IFN-gamma expression by effector and central memory CD4**^**+ **^**T cell in cured ML subjects.** Graph showing the frequency of cells positive for IFN-gamma in the two populations of memory CD4^+^ T cells with or without SLA stimulus from 7 cured patients. The expression of IFN-gamma was determined by flow cytometry. Cells were evaluated in the CD4^+^CD45RA^-^ gate.

The duration that the immunologic response to *Leishmania* antigen remains after therapy may be an indicator of memory duration after infection with *Leishmania* and this information will be relevant in vaccine development for leishmaniasis. Studies using experimental models have shown that even after elimination of *Leishmania* parasites from the host, central memory CD4+ T cells confers protection to re-infection [8]. We assessed memory CD4+ T cell populations in CL and ML cured individuals (Figure 2A,B and C). There was no difference in the percentage of CD4+ effector or central T cells memory populations observed in CL and ML cured patients (p = 0.1). Because the majority of memory T cell populations in humans are not *Leishmania*-specific, we decided to assess the SLA-induced IFN-gamma production by memory T cell populations in CL and ML cured individuals who remained with an immune response as assessed by IFN-gamma production after therapy. The CD4+ T cells are the main IFN-gamma secreting population in those individuals who responded to SLA (data not shown). Moreover, effector memory (CD45RA^-^ CCR7^-^) CD4+ T cells were the main source of IFN-gamma after therapy in subjects whom remained producing IFN-gamma after therapy (p = 0.01) (Figure 3).

## Discussion

The systemic and tissue immunologic response in patients with CL or ML due to *L. braziliensis* have been well characterized and it has been shown that CD4+ T cells are the main source of IFN-gamma and also an important source of TNF-alpha during active disease [3,4]. As only 5% of subjects cured of *L. braziliensis* infection have a new episode of the disease [10] it is likely that protective immunity is maintained long after cure of CL and ML. However, very little is known about memory T cells in human leishmaniasis. A few studies have evaluated immunologic response by lymphocyte proliferation and cytokine production in subjects cured of CL and usually evidence of T cells response have been documented after cure [18-20]. Here we show that the majority of CL and ML cured patients not only decrease IFN-gamma and TNF-alpha production after cure, but also that in a large percentage of cured subjects the IFN-gamma, TNF-alpha and IL-2 was not detected.

Several possibilities may explain the discordant results regarding immunologic response after therapy of leishmaniasis: 1) the kind of antigen used in the assay; 2) if the same antigen was used before and after cure; 3) the antimony dose used for treatment of active lesions; 4) the healing time of the lesions; 5) if the evaluation pre and post therapy was performed in the same subjects. In some of these previous studies, killed or disrupted *L.braziliensis* were used as source of antigen [18-20] and in such case lymphocyte immune response can be observed even in unexposed individuals. Similar levels of IFN-gamma and lower levels of IL-4 and IL-10 were observed in subjects cured of CL in comparison with the concentration of these cytokines in patients with active disease, but the evaluation after therapy was not performed in same subjects [19]. Another study show that in cured CL and ML subjects, IFN-gamma levels were quite variable and in more than half of the patients studied low levels of IFN-gamma were observed [20]. Indeed, despite the recommended dose of pentavalent antimony to treat CL is 20 mg/Kg/day for 20 days, lower dose of intravenous antimony, different duration of treatment and topical rather than intravenous antimony therapy have been used for the treatment of leishmaniasis [21,22]. The time between the initiation of the antimony therapy and the clinical cure is also key in understand the significance of the values obtained for immunological tests after cure. It is known that the majority of patients with CL will have a self-healing disease that is dependent of the duration of the illness. If no information about the time of cure is given, it cannot be ruled out that cure may have occurred independent of the therapy. It is known that individuals with self-healing CL remain with a very strong lymphocyte proliferative response to *Leishmania* antigens [23]. In the present study soluble rather than disrupted *L. braziliensi*s antigen was used, the same dose of antimony was given to all patients, the criteria of cure was standardized and the time between the initiation of therapy and the cure was determined. Therefore it is likely that the presence of one or more of these factors may explain discordant results regarding immunological response after cure of tegumentary leishmaniasis.

As the majority of the cured CL and ML patients had no evidence of IFN-gamma and IL-2 production, the LST was performed in these cured subjects to evaluated *in vivo* immune response to parasite antigen. As a good correlation between *in vitro* immunological response and the DTH reaction is usually observed, the documentation that the LST was positive after therapy in subjects who did not have evidence *in vitro* of T cell response was unexpected. However, a discordance between *in vivo* and *in vitro* immunological tests have been documented in subjects with latent tuberculosis [16,17] and more recently we also observed only a weak concordance between the LST and IFN-gamma production to SLA in household contacts of CL patients [24]. Several possibilities may explain these discordant results such as presence of soluble factors in patients with active disease that may act *in vivo*, the nutritional status of the subjects and sub-populations of memory T cells [25]. As in our case subjects were disease free and well-nourished, it is likely that the positive LST in the absence of immunological response *in vitro* was due to the lack of circulating antigen-specific T cells. In such case as the antigen injected intradermal remain for some period of time in the tissue, there was enough time for memory cells retained in lymph nodes or other tissues migrate to the site of the antigen administration resulting in positive LST.

Our results pointed out that the effector memory (CD45RA^-^ CCR7^-^) CD4+ T cells were the main population producing IFN-gamma in cured CL and ML individuals who responded *in vitro* to SLA. Giving support to our data, previous observation in mouse model of *L. major* infection indicates that effector memory CD4+ T cells are the main source of IFN-gamma [8]. Also, it was shown that in subjects with history of CL caused by *L. major* or *L. tropica*, effector memory CD4+ T cells were the main source of IFN-gamma production [26].

According to the available data in the literature and our current results demonstrating that, even losing circulating *Leishmania* antigen-specific IFN-gamma producers T cell populations, the majority of these individuals do not develop disease. Thus, four main hypothesis can be raised: 1) Individuals that lost circulating IFN-gamma CD4+ T cells have cleared infection and have not been re-infected. 2) Memory CD4+ T cells are confined to lymph nodes rather than in peripheral blood. 3) Memory CD4+ T cells are allocated in skin 4) Resistance to leishmaniasis does not rely on memory CD4+ T cells. All of four possibilities are difficult to be addressed in human studies. However, the hypothesis of memory CD4+ T cells be sequestrated in lymphoid organs or in the skin was previously documented [27-29]. The observation of positive LST in the absence of IFN-gamma producing T cells in response to *Leishmania* antigens argue in favor of the presence of tissue-resident memory T cell (TRM). Actually, it has been shown that TRM may remain resident in the skin even the absence of antigen stimulation and are able to mediate protection to herpes simplex virus infection in C57BL/6 mice [28,29].

As all patients were cured remain question, why some patients have ability to produce IFN-gamma and others loose it. Persistence or not of the parasite after therapy could influence presence or absence of IFN-gamma production. Actually, there is evidence that *Leishmania* may persist in the scars after cure. However, due to ethical reasons biopsies were not performed after cure in this study. Enhancement of IL-10, an important regulatory cytokines has been observed after cure of CL [20]. Moreover, presence of IL-10-producing T cells in dermis of chronic *L. major*-infected mice prevented protection against challenge [30]. However, as assessed by ELISA, we could not find IL-10 production in cured CL and ML patients. Finally, it cannot be ruled out the hypothesis of those cured individuals who maintained *in vitro* response to *Leishmania* antigen is due to recent re-infection.

## Conclusion

Our data pointed out that despite the majority of cured CL and ML patients loose ability to produce cytokines upon *in vitro* stimulation of PBMC with *Leishmania* antigens, immunity to *L.braziliensis* infection last long. In those individuals with evidence of *in vitro* immune response effector memory CD4+ T cells were the IFN-gamma producers. Moreover, our data argues in favor that LST is an important test to assess immunity after cure of leishmaniasis and should be used in addition to *in vitro* tests to evaluate immune response to vaccines in human studies.

## Competing interests

The authors declare that they have no competing interests.

## Authors’ contribution

AMC, LPC and PS participated equally in the study design, data analysis and helped in the preparation of the manuscript. AMC, AM, LPC and OB performed experiments. EMC coordinated the study, participated in its design, evaluation of the results and manuscript preparation. All authors read and approved the final version.

## Pre-publication history

The pre-publication history for this paper can be accessed here:

http://www.biomedcentral.com/1471-2334/13/529/prepub
